# Novel 3-Amino-6-chloro-7-(azol-2 or 5-yl)-1,1-dioxo-1,4,2-benzodithiazine Derivatives with Anticancer Activity: Synthesis and QSAR Study

**DOI:** 10.3390/molecules201219821

**Published:** 2015-12-09

**Authors:** Aneta Pogorzelska, Jarosław Sławiński, Kamil Brożewicz, Szymon Ulenberg, Tomasz Bączek

**Affiliations:** 1 Department of Organic Chemistry, Medical University of Gdańsk, Al. Gen. J. Hallera 107, 80-416 Gdańsk, Poland; jaroslaw@gumed.edu.pl (J.S.); kamil@gumed.edu.pl (K.B.); 2 Department of Pharmaceutical Chemistry, Medical University of Gdańsk, Al. Gen. J. Hallera 107, 80-416 Gdańsk, Poland; szymon.ulenberg@gumed.edu.pl (S.U.); tbaczek@gumed.edu.pl (T.B.)

**Keywords:** 1,4,2-benzodithiazines, sulfonamide, QSAR, anticancer activity

## Abstract

A series of new 3-amino-6-chloro-7-(azol-2 or 5-yl)-1,1-dioxo-1,4,2-benzodithiazine derivatives **5a**–**j** have been synthesized and evaluated *in vitro* for their antiproliferative activity at the U.S. National Cancer Institute. The most active compound **5h** showed significant cytotoxic effects against ovarian (OVCAR-3) and breast (MDA-MB-468) cancer (10% and 47% cancer cell death, respectively) as well as a good selectivity toward prostate (DU-145), colon (SW-620) and renal (TK-10) cancer cell lines. To obtain a deeper insight into the structure-activity relationships of the new compounds **5a**–**j** QSAR studies have been applied. Theoretical calculations allowed the identification of molecular descriptors belonging to the RDF (RDF055p and RDF145m in the MOLT-4 and UO-31 QSAR models, respectively) and 3D-MorSE (Mor32m and Mor16e for MOLT-4 and UO-31 QSAR models) descriptor classes. Based on these data, QSAR models with good robustness and predictive ability have been obtained.

## 1. Introduction

1,4,2-Benzodithiazines are very attractive lead structures for designing new compounds as potential pharmaceutical agents. This can be attributed to their wide range of biological activity as well as to their facility for chemical transformation into 2-mercaptobenzenesulfonamides that would not otherwise be easily obtainable.

**Figure 1:**
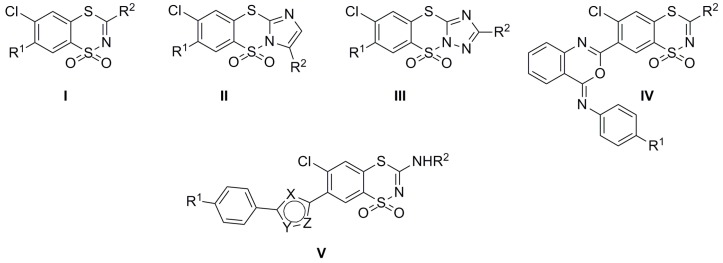
General structures of 1,1-dioxo-1,4,2-benzodithiazines **I**–**IV** [11,12,13,14,15,16,17] and **V** with anticancer activity.

Considering their biological properties, compounds containing a 1,4,2-benzodithiazine scaffold are widely recognized as having a great number of activities, such as diuretic [1,2,3,4,5,6], cholagogue [6,7], radioprotective [4], antiarrhythmic [4,6], hypotensive [4,5,6,7] and anti-HIV [8,9,10]. Of particular interest is that much research on the use of 6-chloro-1,1-dioxo-1,4,2-benzodithiazines as potential therapeutic agents has demonstrated that some of them exhibit remarkable anticancer activity (Figure 1, **I** [11,12,13,14], **II**, **III** [13,15,16] and **IV** [17]). With regard to these reports we have designed novel benzodithiazine derivatives of the general structure of type **V** (Figure 1) that vary according to both the nitrogen-containing 5-membered heterocycle scaffold at position 7 and also the substituent bearing either a condensed indazole or indole ring attached to the amine group at position 3 of the 6-chloro-1,1-dioxo-1,4,2-benzodithiazine ring. These modifications were selected not only based on the biological properties of benzodithiazines but also the significant pharmacological importance of heterocycles with a high nitrogen content.

Thus, herein we report the synthesis and anticancer activity of the new series of 3-(R^2^-amino)-7-(azol-2 or 5-yl)-6-chloro-1,1-dioxo-1,4,2-benzodithiazines (**V**, Figure 1). The new compounds have been investigated for *in vitro* activity against 60 human cancer cell lines from different organs of origin. To correlate the chemical structure of compounds with their potency to inhibit the growth of cancer cells quantitative structure-activity relationship (QSAR) analysis was applied. As a result, the most important parameters controlling the biological properties have been determined using statistical approaches.

## 2. Results

### 2.1. Chemistry

**Scheme 1:**
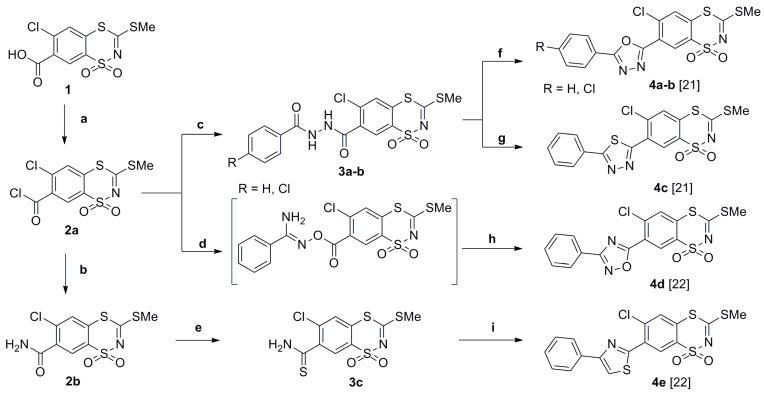
Synthesis of 7-(azol-2 or 5-yl)-6-chloro-3-methylthio-1,1-dioxo-1,4,2-benzodithiazines **4a**–**e**. Reagents and conditions: (**a**) SOCl_2_ excess, benzene, reflux; (**b**) 12% NH_3_(aq), benzene 5–10 °C; (**c**) arylhydrazide, benzene, 5→20 °C; (**d**) *N*-hydroxybenzamidine, toluene, 0→20 °C; (**e**) Lawesson reagent (LR, 0.5 equiv.), toluene; (**f**) SOCl_2_, rt→reflux; (**g**) LR, toluene, 20→100 °C; (**h**) 110 °C; (**i**) ω-halogenoacetophenone, MeOH, 20→65 °C.

**Scheme 2:**
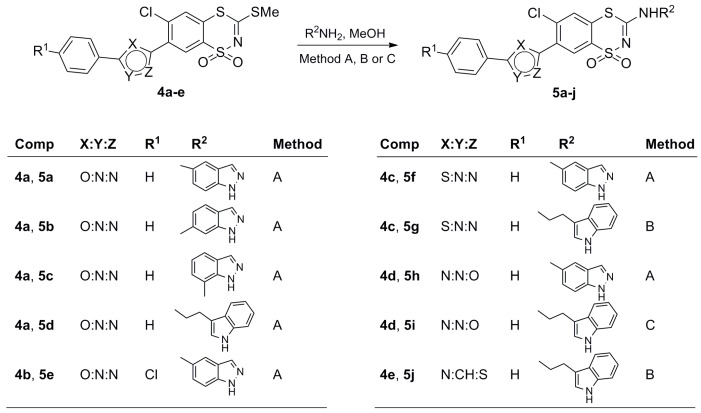
Synthesis of 3-amino-6-chloro-7-(azol-2 or 5-yl)-1,1-dioxo-1,4,2-benzodithiazine derivatives **5a**–**j**. *Reagents and Conditions*: (**A**) reflux, 30–50 h; (**B**) 24 h at room temperature, then reflux, 7.5–48 h; (**C**) room temperature, 52 h.

The synthetic routes for the preparation of the desired 3-(R^2^-amino)-7-(azol-2 or 5-yl)-6-chloro-1,1-dioxo-1,4,2-benzodithiazine derivatives **5a**–**j** are shown in Scheme 1 and Scheme 2.

The essential substrates for the synthesis of novel compounds **5a**–**j**, the already known 6-chloro-7-heteroaryl-3-methylthio-1,1-dioxo-1,4,2-benzodithiazines **4a**–**e**, were obtained using previously described methods which are briefly summarized in Scheme 1 [18,19,20,21,22]. Thus, the starting 3-methylthio-1,1-dioxo-1,4,2-benzodithiazines **4a**–**e** could be converted to the desired 3-(R^2^-amino-7-azolyl-6-chloro-1,1-dioxo-1,4,2-benzodithiazine derivatives **5a**–**j** by nucleophilic substitution reactions with one molar equivalent of a primary amine, as outlined in Scheme 2.

The structures of the final compounds **5a**–**j** were confirmed by elemental analyses and spectroscopic (IR, ^1^H-NMR and ^13^C-NMR) data, in particular the presence of the IR absorption bands corresponding to the stretching vibration of the NH group in the 3228–3408 cm^−1^ range as well as two singlet signals in the ^1^H-NMR spectra related to the NH protons, one in the 10.04–11.79 ppm range for the proton of the NH group attached directly at the position 3 of the benzodithiazine scaffold and another at 10.89–13.24 ppm for the NH linked to the indazole or indole ring (see Supplementary Material, Figures S1–S16).

### 2.2. Anticancer Activity

Compounds **5a**–**j** were tested *in vitro* at the U.S. National Cancer Institute (Bethesda, MD, USA) at a single dose of 10 μM against the NCI panel of 60 cell lines derived from nine different types of cancer, including leukemia, non-small-cell lung cancer (NSCLC), colon, central nervous system (CNS), melanoma, ovarian, renal, prostate and breast. The results, obtained as an inhibition growth percent (IGP), are shown in Table 1. The control was performed via the comparison with no-drug cell growth.

The best anticancer activity was noticed for compounds **5a** and **5h**. The derivative **5a** has inhibited the growth of 21 cancer cell lines with mean IGP value at 19.33%. In the case of **5h** the activity against 28 cancer cell lines has been observed with mean IGP at 43.93%.

### 2.3. QSAR Studies

To further elucidate the structure-activity relationships among the series of compounds **5a**–**j** QSAR methodology was applied. Statistical analysis is essential for QSAR work and therefore, in order to avoid statistical outliers, we selected cancer cell lines which were sensitive toward all studied derivatives **5a**–**j** (0% < IGP < 100%)—leukemia MOLT-4 and renal cancer UO-31. In presented study, the QSAR models for each cell line were developed separately. Descriptors have been calculated using DRAGON, SPARTAN and Gaussian software. After excluding descriptors with no variation or value and adding descriptors from Spartan software and dihedral angle values, a final set comprised of 2765 molecular descriptors. Using Data Mining feature selection, only 490 most statistically important descriptors were chosen for further analysis. After importing the previously mentioned set of descriptors into STATISTICA 10.0 software (Statsoft, Tulsa, OK, USA) Stepwise Multiple Linear Regression (MLR) was performed. The IGP was taken as a dependent value for analysis, while molecular descriptors were independent values. Achieved models were validated using Leave-One-Out Cross-validation and the results are presented in Table 2. Molecular descriptors that entered the model, along with their meaning and values have been presented in Table 3.

**Table 1:** The inhibition growth percent of selected NCI-60 cancer cells (IGP) at a single concentration of 10^−5^ M of novel 1,4,2-benzodithiazine derivatives **5a**–**j**.

Panel	Cell Line	IGP (%) of Compound									
		5a	5b	5c	5d	5e	5f	5g	5h	5i	5j
*Leukemia*	CCRF-CEM	31.62	11.56	-	-	3.41	-	25.24	13.73	6.77	10.91
	HL-60(TB)	31.12	36.36	4.20	*	*	*	*	69.38	*	*
	MOLT-4	16.23	17.07	4.05	12.96	3.64	10.64	23.59	9.06	0.44	24.25
	RPMI-8226	24.20	2.85	*	-	*	0.99	18.21	81.00	1.18	*
	SR	-	-	-	21.80	*	53.71	26.54	1.76	6.36	22.08
*NSCLC*	HOP-62	15.32	13.98	*	*	*	6.75	*	34.63	*	*
	NCI-H226	13.09	26.50	6.62	*	*	17.09	3.34	14.78	1.10	*
	NCI-H522	-	-	15.60	*	5.67	22.52	5.34	27.30	*	*
*Colon cancer*	HCC-2998	-	-	*	*	*	5.10	*	60.93	*	*
	HCT-116	9.47	2.28	*	0.68	*	3.30	7.37	43.60	*	8.42
	SW-620	*	9.09	*	*	*	*	*	50.98	*	*
*CNS cancer*	SNB-19	6.57	9.45	*	4.26	*	17.47	9.70	3.40	2.53	*
	SNB-75	7.09	-	-	11.44	4.47	-	32.19	76.93	12.31	8.52
	U251	12.89	16.48	*	*	*	17.55	5.97	21.68	*	*
*Melanoma*	LOX IMVI	3.56	10.33	*	2.14	*	1.30	8.79	53.68	3.02	*
	UACC-257	11.40	1.35	*	*	*	*	*	33.69	*	*
	UACC-62	16.61	*	*	4.08	*	5.83	0.29	23.80	2.39	*
*Ovarian cancer*	OVCAR-3	*	7.74	*	*	*	*	*	10.27 ^a^	*	*
	OVCAR-8	15.93	15.64	*	2.07	*	0.66	8.81	24.08	1.69	*
*Renal cancer*	SN12C	2.62	14.33	1.69	4.46	*	17.11	11.87	15.54	1.69	*
	TK-10	*	*	5.41	*	*	*	*	52.33	*	*
	UO-31	4.78	36.34	16.35	23.53	8.75	34.00	29.54	6.57	24.73	9.74
*Prostate cancer*	PC-3	19.12	11.45	1.37	-	6.74	*	1.87	51.71	*	0.36
	DU-145	*	*	*	*	*	*	*	98.79	*	*
*Breast cancer*	MCF-7	35.97	6.77	8.25	4.44	*	10.50	15.78	23.52	5.32	10.35
	MDA-MB-231/ATCC	25.84	15.13	1.75	*	*	*	*	42.05	*	*
	T-47D	49.10	12.27	*	*	*	*	35.19	37.64	*	*
	MDA-MB-468	53.37	18.73	5.58	*	*	9.62	4.93	47.18 ^a^	*	*

* IGP ≤ 0%; - not tested; **^a^**—cytotoxic effect.

**Table 2:** Developed QSAR models and their performance in predicting anticancer activity of 3-amino-6-chloro-7-(azol-2 or 5-yl)-1,1-dioxo-1,4,2-benzodithiazine derivatives **5a**–**j** against MOLT-4 and UO-31 cell lines.

Cell Line	Equation	N	R	R_cv_	s	RMSECV	*p*	F
MOLT-4	IGP = 0.815(RDF055p) − 0.55(Mor32m) − 41.34	10	0.967	0.927	2.34	0.859	0.00006	51.34
UO-31	IGP = 0.770(Mor16e) − 0.497(RDF145m) + 67.048	10	0.943	0.861	11.44	1.371126	0.0006	28.33

N—number of compounds; R—correlation coefficient; R_cv_—correlation coefficient of leave-one-out cross-validation (LOO-CV); s—a standard error of estimate; RMSECV—a root mean square error LOO-CV, *p*—significance level of F-test; F—Fisher test value

**Table 3:** Molecular descriptors along with their values and interpretation used for developing QSAR equations. **RDF055p**—Radial Distribution Function—055/weighted by polarizability; **Mor32m**—signal 32/weighted by mass; **Mor16e**—signal 16/weighted by Sanderson electronegativity; **RDF145m**—Radial Distribution Function—145/weighted by mass.

Compd.	MOLT-4		UO-31	
	RDF055p	Mor32m	Mor16e	RDF145m
**5a**	5.903	−0.701	1.632	3.349
**5b**	6.251	−0.775	1.243	5.546
**5c**	4.882	−0.627	1.529	3.010
**5d**	5.670	−0.616	1.744	5.816
**5e**	6.278	−0.202	1.765	3.392
**5f**	6.038	−0.486	1.252	3.104
**5g**	8.458	−0.549	1.260	3.370
**5h**	5.958	−0.448	1.662	1.985
**5i**	5.521	−0.400	1.289	3.139
**5j**	7.891	−0.551	1.565	1.986

Good correlation between the data obtained by *in vitro* studies and the one predicted by QSAR model application has been obtained as presented in Table 4.

**Table 4:** Comparison of observed IGP values (anticancer activity) and those predicted by QSAR models for MOLT-4 and UO-31 cell lines.

Compd.	IGP [%]			
	MOLT-4		UO-31	
	Observed	Predicted	Observed	Predicted
**5a**	16.23	13.82	4.78	14.44
**5b**	17.07	19.33	36.34	43.11
**5c**	4.05	6.96	16.35	15.78
**5d**	12.96	10.15	23.53	11.71
**5e**	3.64	2.13	8.75	7.11
**5f**	10.64	9.13	34	25.97
**5g**	23.59	28.91	29.54	28.72
**5h**	9.06	7.55	6.57	5.05
**5i**	0.44	4.77	24.73	27.15
**5j**	24.25	21.63	9.74	9.61

## 3. Discussion

### 3.1. Anticancer Activity

Considering the anticancer activity results (see Table 1) it has been observed that the nature and structure of substituents located at positions 3 and 7 of benzodithiazine have varying influences on the compounds’ anticancer activity. However, the most important features seem to be both the electronic character of the substituent at the position 7 and the substitution pattern of the heterocyclic ring attached to the amine group in position 3.

Derivative **5h**, possessing a 3-phenyl-1,2,4-oxadiazol-5-yl moiety in position 7 as well as a 1*H*-indazol-5-yl scaffold attached directly to the amine group in position 3, shows the best anticancer properties. Particularly noteworthy is the cytotoxic effect observed against ovarian cancer OVCAR-3 and breast cancer MDA-MB-468. Moreover, only compound **5h** displayed the ability to inhibit growth of the DU-145 prostate cancer cell line at a level of 98.79%. A similar, although slightly lower, selectivity has been observed in the case of colon cancer SW-620 (IGP = 50.98%) and renal cancer TK-10 (IGP = 52.33%).

Good anticancer properties have been observed for compounds bearing a 5-phenyl-1,3,4-oxadiazol-2-yl scaffold at position 7 of the benzodithiazine and either 1*H*-indazol-5-yl (**5a**) or 1*H*-indazol-6-yl (**5b**) fragments in position 3. On the other hand, a significant decrease in activity was observed after incorporation of substituents such as (1*H*-indazol-7-yl)amino (**5c**) or 2-(1*H*-indol-3-yl)ethylamino (**5d**) at position 3. These findings prove the importance of the structure in this position. However, in the case of compounds **5e** and **5f** bearing 5-phenyl-1,3,4-thiadiazol-2-yl at position 7 (instead of an oxadiazole ring), modification of position 3 seems to have much lower impact. The influence of the electronic character of the substituent located at position 7 of the benzodithiazine scaffold could be especially observed in the case of derivative **5e**. The replacement of 5-phenyl-1,3,4-oxadiazol-2-yl (**5a**) by 5-(4-chlorophenyl)-1,3,4-oxadiazol-2-yl (**5e**) led to an almost completely lack of anticancer activity.

### 3.2. QSAR Studies

The application of QSAR methodology led to the obtaining of descriptors for building QSAR models which provide not only information useful for further chemical synthesis, but also enable the prediction of pharmacological activity of novel derivatives. It should be emphasized that developed models showed good correlation of chosen descriptors with activity of compounds. Moreover QSAR models for both MOLT-4 and UO-31 cell lines showed good predictability, as presented in Table 2.

Difficulty in practical interpretation of model descriptors for both cell lines shows the complex nature of mode of action of the studied derivatives. Further study on a larger set of compounds might reveal structure-activity relationships that are easier to interpret, providing valuable guidelines for further synthesis. However, when comparing observed anticancer activity with that predicted by statistical analysis (Table 4), the developed models already show good performance and prove to be useful in this kind of study

## 4. Experimental Section

### 4.1. General Information

The melting points were determined on a Boethius PHMK apparatus (Veb Analytic, Dresden, Germany) and are uncorrected. Infrared (IR) spectra were taken on a Thermo Mattson Satellite FTIR spectrophotometer (Thermo Mattson, Madison, WI, USA). The NMR spectra were recorded on a Varian Gemini 200 apparatus (Varian, Palo Alto, CA, USA) at 200 MHz (^1^H-NMR) and 50 MHz (^13^C-NMR) or on a Varian Unity 500 Plus apparatus (Varian, Palo Alto, CA, USA) at 500 MHz (^1^H-NMR) and 125 MHz (^13^C-NMR). Chemical shifts are expressed as δ values in parts per million (ppm) relative to TMS as an internal standard. Spectra were acquired in deuterated dimethylsulfoxide (DMSO-*d*_6_). The results of elemental analyses for C, H and N were in agreement with the theoretical values within ±0.4% range. The commercially unavailable substrates were obtained according to the following previously described methods : 6-chloro-3-methylthio-1,1-dioxo-1,4,2-benzodithiazine-7-carboxylic acid (**1**) [18], 6-chloro-3-methylthio-1,1-dioxo-1,4,2-benzodithiazine-7-carbonyl chloride (**2a**) [19], 6-chloro-3-methylthio-1,1-dioxo-1,4,2-benzodithiazine-7-carboamide (**2b**) [20], *N′*-(6-chloro-3-methylthio-1,1-dioxo-1,4,2-benzodithiazine-7-carbonyl)benzhydrazides (**3a**–**b**) [21], 6-chloro-7-(5-aryl-1,3,4-oxa or 1,3,4-thiadiazol-2-yl)-3-methylthio-1,1-dioxo-1,4,2-benzodithiazines (**4a**–**c**) [21], 7-(3-phenyl-1,2,4-oxadiazol-5-yl)-6-chloro-3-methylthio-1,1-dioxo-1,4,2-benzodithiazine (**4d**) [22] and 7-(4-phenylthiazol-2-yl)-6-chloro-3-methylthio-1,1-dioxo-1,4,2-benzodithiazine (**4e**) [22]. The NMR spectra of newly synthesized compounds **5a**–**j** have been given as Figures S1–S16 in Supplementary Material.

### 4.2. Synthesis

#### General Procedure for the Preparation of 3-(R^2^-Amino)-7-(azol-2 or 5-yl)-6-chloro-1,1-dioxo-1,4,2-benzodithiazines **5a**–**j**

To a suspension of the 7-(azol-2 or 5-yl)-6-chloro-3-methylthio-1,1-dioxo-1,4,2-benzodithiazine **4a**–**e** (1.0 mmol) in methanol (10 mL) the appropriate primary amine (1.0 mmol) was added. The reaction mixture was refluxed for 30–50 h (method A—**5a**–**f**, **5h**), stirred for 24 h at room temperature and then refluxed for 7.5–48 h (method B—**5g**, **5j**) or stirred at room temperature for 52 h (method C—**5i**) until the methanethiol was released. The precipitated solid was filtered off and washed several times with methanol. The crude product was purified by crystallization from the appropriate solvent.

*6-Chloro-7-(5-phenyl-1*,*3*,*4-oxadiazol-2-yl)-3-[(1H-indazol-5-yl)amino]-1*,*1-dioxo-1*,*4*,*2-benzodithiazine* (**5a**). Starting from **4a** (0.424 g) and 5-amino-1*H*-indazole (0.133 g) after refluxing for 48 h the title compound **5a** was obtained (0.443 g, 87%), mp 325–328 °C (DMF–MeOH, 1:3); IR (KBr) ν_max_ 3387, 3296 (NH), 2925, 2854 (CH), 1589, 1548, 1531, 1505, 1450 (C=N, C=C), 1312, 1159 (SO_2_) cm^−1^; ^1^H-NMR (200 MHz, DMSO-*d*_6_) δ 7.50–7.60 (m, 1H, Ar H), 7.62–7.69 (m, 4H, Ar H), 8.11–8.18 (m, 4H, Ar H), 8.37 (s, 1H, H-5), 8.63 (s, 1H, H-8), 11.67 (s, 1H, NH), 13.22 (s, 1H, NH) ppm; anal. C 51.92, H 2.57, N 16.51% calcd for C_22_H_13_ClN_6_O_3_S_2_, C 52.03 H 2.85 N 16.80%.

*6-Chloro-7-(5-phenyl-1*,*3*,*4-oxadiazol-2-yl)-3-[(1H-indazol-6-yl)amino]-1*,*1-dioxo-1*,*4*,*2-benzodithiazine* (**5b**). Starting from **4a** (0.424 g) and 6-amino-1*H*-indazole (0.133 g) after refluxing for 30 h the title compound **5b** was obtained (0.244 g, 48%) mp 336–338 °C (DMF–MeOH, 1:3); IR (KBr) ν_max_ 3288 (NH), 2923 (CH), 1632, 1609, 1588, 1539, 1476, 1450 (C=N, C=C), 1305, 1160 (SO_2_) cm^−1^; ^1^H-NMR (500 MHz, DMSO-*d*_6_) δ 7.23–7.24 (m, 1H, Ar H), 7.61–7.63 (m, 3H, Ar H), 7.79–7.80 (m, 1H, Ar H), 8.07–8.14 (m, 4H, Ar H), 8.32 (s, 1H, H-5), 8.62 (s, 1H, H-8), 11.77 (s, 1H, NH), 13.17 (s, 1H, NH) ppm; ^13^C-NMR (125 MHz, DMSO-*d*_6_) δ 102.62, 115.50, 121.23, 122.91, 122.97, 126.37, 126.90, 129.50, 130.92, 130.98, 132.39, 133.60, 133.84, 135.09, 135.54, 139.76, 160.88, 164.57 ppm; anal. C 51.92, H 2.57, N 16.51% calcd for C_22_H_13_ClN_6_O_3_S_2_, C 52.12, H 2.63, N 16.75%.

*6-Chloro-7-(5-phenyl-1*,*3*,*4-oxadiazol-2-yl)-3-[(1H-indazol-7-yl)amino]-1*,*1-dioxo-1*,*4*,*2-benzodithiazine* (**5c**). Starting from **4a** (0.424 g) and 7-amino-1*H*-indazole (0.133 g) after refluxing for 50 h the title compound **5c** was obtained (0.224 g, 44%) mp 329–332 (dec.) °C (DMF–MeOH, 1:3); IR (KBr) ν_max_ 3289 (NH), 2927 (CH), 1649, 1581, 1551, 1488, 1459 (C=N, C=C), 1316, 1162 (SO_2_) cm^−1^; ^1^H-NMR (500 MHz, DMSO-*d*_6_) δ 7.18–7.19 (m, 1H, Ar H), 7.40–7.41 (m, 1H, Ar H), 7.62–7.67 (m, 3H, Ar H), 7.78–7.79 (m, 1H, Ar H), 8.11–8.17 (m, 3H, Ar H), 8.40 (s, 1H, H-5), 8.61 (s, 1H, H-8), 11.69 (s, 1H, NH), 13.12 (s, 1H, NH) ppm; ^13^C-NMR (125 MHz, DMSO-*d*_6_) δ 120.30, 120.44, 122.45, 122.80, 122.95, 126.44, 126.93, 126.98, 129.54, 130.80, 131.33, 132.42, 134.07, 134.22, 135.00, 135.39, 160.90, 164.61 ppm; anal. C 51.92, H 2.57, N 16.51% calcd for C_22_H_13_ClN_6_O_3_S_2_, C 52.03, H 2.91 N 16.60%.

*6-Chloro-7-(5-phenyl-1*,*3*,*4-oxadiazol-2-yl)-3-[2-(1H-indol-3-yl)ethylamino]-1*,*1-dioxo-1*,*4*,*2-benzodithiazine* (**5d**). Starting from **4a** (0.424 g) and 2-(1*H*-indol-3-yl)ethanamine (0.160 g) after refluxing for 30 h the title compound **5d** was obtained (0.278 g, 53%) mp 262–264 °C (DMF–MeOH, 1:3); IR (KBr) ν_max_ 3408 (NH), 3080 (CH Ar), 2925 (CH), 1568, 1488, 1450 (C=N, C=C), 1303, 1159 (SO_2_) cm^−1^; ^1^H-NMR (500 MHz, DMSO-*d*_6_) δ 3.00–3.02 (m, 2H, CH_2_), 3.65–3.68 (m, 2H, CH_2_), 6.95–6.98 (m, 1H, Ar H), 7.06(t, *J* = 7.3 Hz, 1H, Ar H), 7.20 (s, 1H, Ar H), 7.33 (d, *J* = 8.3 Hz, 1H, Ar H), 7.55 (d, *J* = 8.3 Hz, 1H, Ar H), 7.63–7.70 (m, 3H, Ar H), 8.13–8.14 (m, 2H, Ar H), 8.26 (s, 1H, H-5), 8.60 (s, 1H, H-8), 10.04 (s, 1H, NH), 10.89 (s, 1H, NH) ppm; anal. C 56.02, H 3.38, N 13.07% calcd for C_25_H_18_ClN_5_O_3_S_2_, C 56.31, H 3.52, N 13.24%.

*6-Chloro-7-[5-(4-chlorophenyl)-1*,*3*,*4-oxadiazol-2-yl]-3-[(1H-indazol-5-yl)amino]-1*,*1-dioxo-1*,*4*,*2-benzodithiazine* (**5e**). Starting from **4b** (0.458 g) and 5-amino-1*H*-indazole (0.133 g) after refluxing for 44 h the title compound **5e** was obtained (0.212 g, 39%) mp 352–354 (dec.) °C (DMF–MeCN, 3:4); IR (KBr) ν_max_ 3383 (NH), 3079 (CH Ar), 2925, 2854 (CH), 1604, 1530, 1506, 1482, 1459 (C=N, C=C), 1320, 1159 (SO_2_) cm^−1^; ^1^H-NMR (200 MHz, DMSO-*d*_6_) δ 7.66–7.74 (m, 4H, Ar H), 8.09–8.18 (m, 4H, Ar H), 8.37 (s, 1H, H-5), 8.64 (s, 1H, H-8), 11.66 (s, 1H, NH), 13.22 (s, 1H, NH) ppm; anal. C 48.63, H 2.23, N 15.47% calcd for C_22_H_12_Cl_2_N_6_O_3_S_2_, C 48.95, H 2.39 N 15.62%.

*6-Chloro-7-(5-phenyl-1*,*3*,*4-thiadiazol-2-yl)-3-[(1H-indazol-5-yl)amino]-1*,*1-dioxo-1*,*4*,*2-benzodithiazine* (**5f**). Starting from **4c** (0.440 g) and 5-amino-1*H*-indazole (0.133 g) after refluxing for 45 h the title compound **5f** was obtained (0.415 g, 79%) mp >360 °C (DMF–MeOH, 1:3); IR (KBr) ν_max_ 3299 (NH), 2925, 2853 (CH), 1611, 1575, 1531, 1504, 1457 (C=N, C=C), 1305, 1157 (SO_2_) cm^−1^; ^1^H-NMR (500 MHz, DMSO-*d*_6_) δ: 7.50–7.52 (m, 1H, Ar H), 7.57–7.62 (m, 5H, Ar H), 8.03–8.10 (m, 2H, Ar H), 8.16 (s, 1H, Ar H), 8.29 (s, 1H, H-5), 8.76 (s, 1H, H-8), 11.67 (s, 1H, NH), 13.21 (s, 1H, NH) ppm; ^13^C-NMR (125 MHz, DMSO-*d*_6_) δ 111.42, 114.58, 122.61, 123.21, 126.41, 128.48, 129.46. 129.69, 130.26, 131.08, 132.14, 132.45, 133.64, 134.76, 135.37, 138.55, 160.63, 161.96, 170.41 ppm; anal. C 50.33, H 2.50 N 16.01% calcd for C_22_H_13_ClN_6_O_2_S_3_, C 50.52, H 2.81 N 15.95%.

*6-Chloro-7-(5-phenyl-1*,*3*,*4-thiadiazol-2-yl)-3-[2-(1H-indol-3-yl)ethylamino]-1*,*1-dioxo-1*,*4*,*2-benzodithiazine* (**5g**). Starting from **4c** (0.440 g) and 2-(1*H*-indol-3-yl)ethanamine (0.160 g) after stirring for 24 h in room temperature followed by refluxing for 7.5 h the title compound **5g** was obtained (0.488 g, 93%) mp 227–229 °C (DMF–MeOH, 1:3); IR (KBr) ν_max_ 3228 (NH), 2962, 2929, 2873 (CH), 1649, 1602 1575, 1486, 1452 (C=N, C=C), 1316, 1164 (SO_2_) cm^−1^; ^1^H-NMR (500 MHz, DMSO-*d*_6_) δ 3.01 (m, 2H, CH_2_), 3.67 (m, 2H, CH_2_), 6.97–6.98(m, 1H, Ar H), 7.04–7.07 (m, 1H, Ar H), 7.20 ( s, 1H, Ar H), 7.33–7.34 ( m, 1H, Ar H), 7.55–7.57 (m, 4H, Ar H), 8.04–8.05 (m, 2H, Ar H), 8.19 (s, 1H, H-5), 8.74 (s, 1H, H-8), 10.02 (s, 1H, NH), 10.89 (s, 1H, NH) ppm; ^13^C-NMR (125 MHz, DMSO-*d*_6_) δ 23.80, 44.28, 110.73, 111.45, 118.17, 118.40, 121.06, 122.99, 125.59, 127.04, 127.78, 128.50, 129.02, 129.57, 130.13, 131.75, 132.01, 132.93, 134.44, 136.24, 161.33, 161.64, 169.67 ppm; anal. C 54.39, H 3.29, N 12.69% calcd for C_25_H_18_ClN_5_O_2_S_3_, C 54.58, H 3.35, N 12.73%.

*6-Chloro-7-(3-phenyl-1*,*2*,*4-oxadiazol-5-yl)-3-[(1H-indazol-5-yl)amino]-1*,*1-dioxo-1*,*4*,*2-benzodithiazine* (**5h**). Starting from **4d** (0.424 g) and 5-amino-1*H*-indazole (0.133 g) after refluxing for 50 h the title compound **5h** was obtained (0.341 g, 67%) mp 347–349 °C (70% DMF_aq_); IR (KBr) ν_max_ 3385 (NH), 1595, 1565, 1531 (C=N, C=C), 1321, 1158 (SO_2_) cm^−1^; ^1^H-NMR (500 MHz, DMSO-*d*_6_) δ 7.55 (m, 1H, Ar H), 7.62–7.66 (m, 4H, Ar H), 8.11 (s, 1H, Ar H), 8.14–8.15 (m, 2H, Ar H), 8.19 (s, 1H, Ar H), 8.41 (s, 1H, H-5), 8.71 (s, 1H, H-8), 11.75 (s, 1H, NH), 13.24 (s, 1H, NH) ppm; anal. C 51.92, H 2.57, N 16.51% calcd for C_22_H_13_ClN_6_O_3_S_2_, C 52.08, H 2.69, N 16.62%.

*6-Chloro-7-(3-phenyl-1*,*2*,*4-oxadiazol-2-yl)-3-[2-(1H-indol-3-yl)ethylamino]-1*,*1-dioxo-1*,*4*,*2-benzodithiazine* (**5i**). Starting from **4d** (0.424 g) and 2-(1*H*-indol-3-yl)ethanamine (0.160 g) after stirring for 52 h in room temperature the title compound **5i** was obtained (0.306 g, 57%) mp 205–207 °C (MeOH); IR (KBr) ν_max_ 3403, 3283 (NH), 3049 (CH Ar), 2921, 2854 (CH), 1599, 1567, 1475, 1450 (C=N, C=C), 1314, 1141 (SO_2_) cm^−1^; ^1^H-NMR (500 MHz, DMSO-*d*_6_) δ 3.00–3.03 (m, 2H, CH_2_), 3.66–3.69 (m, 2H, CH_2_), 6.95–6.98 (m, 1H, Ar H), 7.05–7.08 (m, 1H, Ar H), 7.20 ( s, 1H, Ar H), 7.34 (d, *J* = 8.3 Hz, 1H, Ar H), 7.54–7.58 (m, 1H, Ar H), 7.60–7.62 (m, 3H, Ar H), 8.09–8.10 (m, 2H, Ar H), 8.24 (s, 1H, H-5), 8.64 (s, 1H, H-8), 10.07 (s, 1H,NH), 10.89 (s, 1H, NH) ppm; ^13^C-NMR (125 MHz, DMSO-*d*_6_) δ 24.44, 45.02, 111.40, 112.14, 118.85, 119.09, 121.74, 123.44, 123.69, 126.37, 127.73, 127.89, 127.91, 130.04, 131.50, 132.48, 132.57, 135.91, 136.07, 136.92, 162.13, 168.71, 173.28 ppm; anal. C 56.02, H 3.38, N 13.07% calcd for C_25_H_18_ClN_5_O_3_S_2_, C 56.38, H 3.50,N 13.31%.

*6-Chloro-7-(4-phenylthiazol-2-yl)-3-[2-(1H-indol-3-yl)ethylamino]-1*,*1-dioxo-1*,*4*,*2-benzodithiazine* (**5j**). Starting from **4e** (0.439 g) and 2-(1*H*-indol-3-yl)ethanamine (0.160 g) after stirring for 24 h in room temperature followed by refluxing for 48 h the title compound **5j** was obtained (0.518 g, 94%) mp 145–147 °C (MeOH); IR (KBr) ν_max_ 3408 (NH), 2925, 2853 (CH), 1563, 1458 (C=N, C=C), 1306, 1156 (SO_2_) cm^−1^; ^1^H-NMR (500 MHz, DMSO-*d*_6_) δ 3.01–3.03 (m, 2H, CH_2_), 3.67 (m, 2H, CH_2_), 6.96–6.99 (m, 1H, Ar H), 7.05–7.08 (m, 1H, Ar H), 7.20 (s, 1H, Ar H), 7.33–7.40 (m, 2H, Ar H), 7.47–7.50 (m, 2H, Ar H), 7.55–7.57 (m, 1H, Ar H), 8.03–8.04 (m, 2H, Ar H), 8.12 (s, 1H, H-5), 8.38 (s, 1H, Ar H), 8.88 (s, 1H, Ar H), 9.98 (s, 1H, NH), 10.89 (s, 1H, NH) ppm; ^13^C-NMR (125 MHz, DMSO-*d*_6_) δ 23.80, 44.24, 110.76,.11.45, 117.64, 118.18, 118.40, 121.06, 122.98, 125.24, 126.19, 127.06, 128.52, 128.95, 130.19, 131.15, 131.99, 133.40, 133.57, 136.24, 154.26, 160.05, 161.76 ppm; anal. C 56.66, H 3.48, N 10.17% calcd for C_26_H_19_ClN_4_O_2_S_3_, C 56.80, H 3.61, N 10.42%.

### 4.3. In Vitro Anticancer Screening

Antitumor evaluation of compounds **5a**–**j** was performed at the National Cancer Institute according to NCI-60 DTP Human Tumor Cell Line Screen procedure [23,24,25,26].

### 4.4. Methodology of Molecular Modeling and QSAR Models Development

Studied compounds were manually drawn using ACD ChemSketch (Advanced Chemistry Development, Inc., Toronto, ON, Canada), and geometrically optimized afterwards. AM1 pre-optimization was conducted using HyperChem (v 8.0.8, HyperCube, Gainesville, FL, USA). DFT calculations were conducted using Gaussian software [27], at the B3LYP/6–311 G(d) level of theory.

Molecular descriptors were calculated using DRAGON 6.0 Software (Talete, Milano, Italy), SPARTAN software (Spartan ’08, Wavefunction, Inc., Irvine, CA, USA) and Gaussian software [27].

Statistical analysis, feature selection and chemometric calculations (Stepwise Multiple Linear Regression) were all conducted using STATISTICA 10.0 software. IGP was taken as a dependent value for analysis, while molecular descriptors were independent values.

Created models were validated using Leave-one-out cross-validation (LOO-CV). This procedure assumed removing single data point (cytotoxic value) from analyzed set, recalculating regression on the rest of the dataset, and comparing predicted cytotoxic value of the omitted compound with experimental value. The procedure was repeated until each compound’s cytotoxic value has been omitted once. To evaluate developed model’s performance, sum of squares of each omitted data errors were used to calculate the cross-validated root-mean-square error (RMSECV).

## 5. Conclusions

We have developed a facile method for the synthesis of new 3-amino-6-chloro-7-(azol-2 or 5-yl)-1,1-dioxo-1,4,2-benzodithiazine derivatives. The compounds were evaluated *in vitro* for their antiproliferative activity at the U.S. National Cancer Institute. We have found that the novel compounds displayed moderate anticancer activity related to their structure. The best antiproliferative properties have been observed for compound **5h**, especially against the ovarian (OVCAR-3) and breast (MDA-MB-468) cancer cell lines. Moreover, good selectivity against prostate (DU-145), colon (SW-620) and renal (TK-10) cancer cell lines have also been observed for derivative **5h**. To summarize the structure-activity relationship very briefly, it could be stated that derivatives possessing a 3-phenyl-1,2,4-oxadiazol-5-yl or 5-phenyl-1,3,4-oxadiazol-2-yl moiety attached directly to position 7 as well as a 1*H*-indazol-5-yl scaffold incorporated in the position 3 display the best anticancer properties. The QSAR studies have revealed that the atomic masses and atomic polarizability weighted descriptors played a significant role in addressing compounds activity against the leukemia (MOLT-4) cell line. On the other hand, atomic masses and atomic Sanderson electronegativity have a greater impact on the anticancer activity toward renal cancer (UO-31) cell line. The comparison of the cytotoxic activity with the one predicted by statistical analysis has shown that the obtained QSAR models display a good correlation with R_cv_ values of 0.927 and 0.861 for MOLT-4 and UO-31 respectively, suggesting these models can be used in order to design new structures with interesting anticancer activity.

## Supplementary Materials

Supplementary materials can be accessed at: http://www.mdpi.com/1420-3049/20/12/19821/s1.
