# Supplementary material for: Novel 3-Amino-6-chloro-7-(azol-2 or 5-yl)-1,1-dioxo-1,4,2-benzodithiazine Derivatives with Anticancer Activity: Synthesis and QSAR Study
Source: Molecules. 2015 Dec 9;20(12):21960–70. doi: 10.3390/molecules201219821 (PMC6332169; doi:10.3390/molecules201219821)
Supplement: Supplementary file 1 [file molecules-20-19821-s001.pdf]

Supplementary Material: Novel 3-Amino-6-chloro-7-(azol-2 or 5-yl)-1,1-dioxo-1,4,2-benzodithiazine Derivatives with Anticancer Activity-Synthesis and QSAR Study

Aneta Pogorzelska, Jarosław Sławiński, Kamil Brożewicz, Szymon Ulenberg and Tomasz Bączek

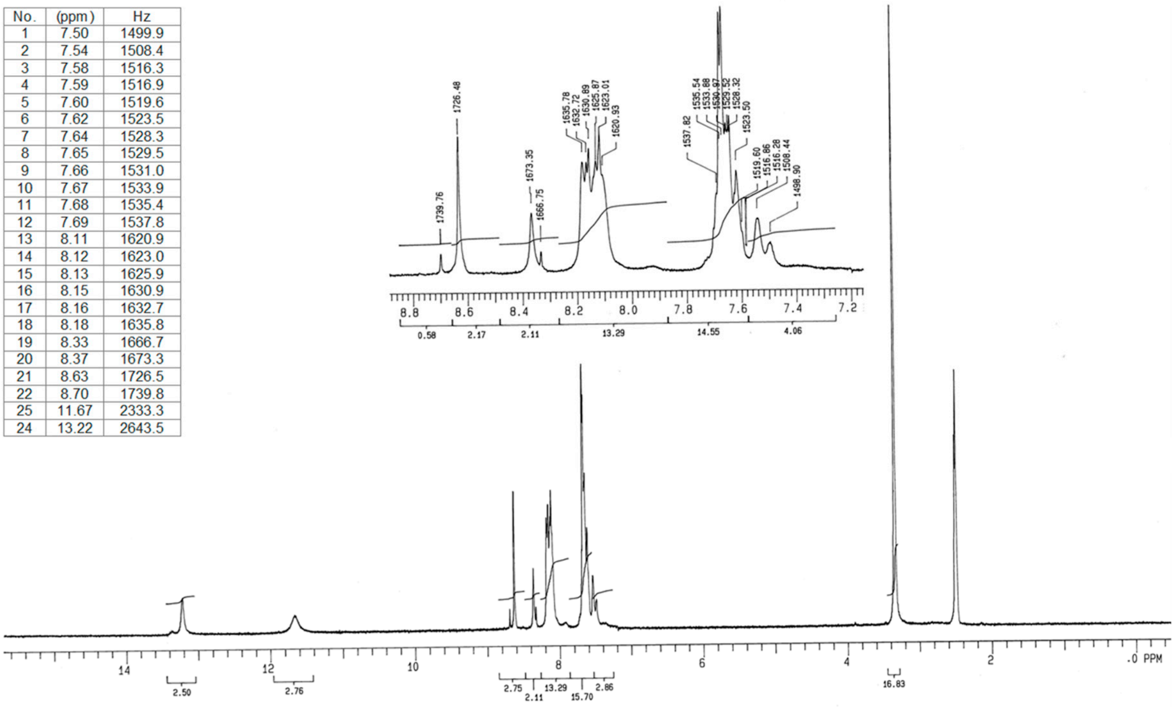

No.	(ppm)	Hz
1	7.50	1499.9
2	7.54	1508.4
3	7.58	1516.3
4	7.59	1516.9
5	7.60	1519.6
6	7.62	1523.5
7	7.64	1528.3
8	7.65	1529.5
9	7.66	1531.0
10	7.67	1533.9
11	7.68	1535.4
12	7.69	1537.8
13	8.11	1620.9
14	8.12	1623.0
15	8.13	1625.9
16	8.15	1630.9
17	8.16	1632.7
18	8.18	1635.8
19	8.33	1666.7
20	8.37	1673.3
21	8.63	1726.5
22	8.70	1739.8
25	11.67	2333.3
24	13.22	2643.5

Figure S1. ^{1}H-NMR of compound **5a** (200 MHz, DMSO-d_6).

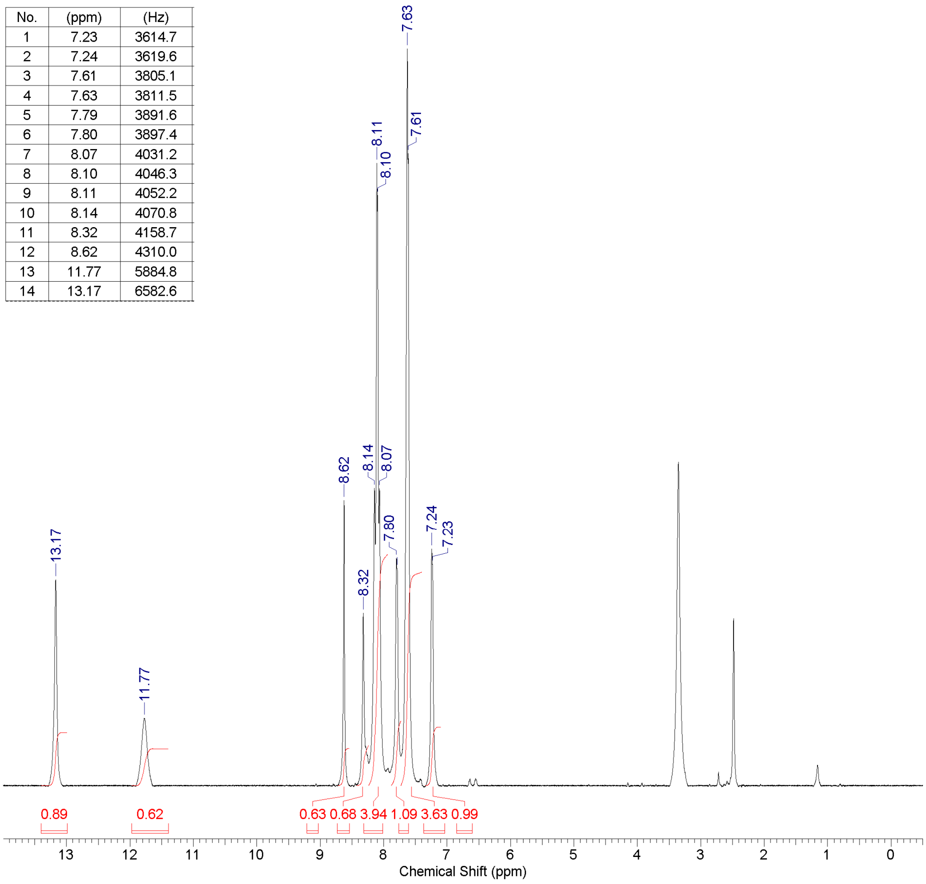

No.	(ppm)	(Hz)
1	7.23	3614.7
2	7.24	3619.6
3	7.61	3805.1
4	7.63	3811.5
5	7.79	3891.6
6	7.80	3897.4
7	8.07	4031.2
8	8.10	4046.3
9	8.11	4052.2
10	8.14	4070.8
11	8.32	4158.7
12	8.62	4310.0
13	11.77	5884.8
14	13.17	6582.6

Figure S2. ^{1}H-NMR of compound **5b** (500 MHz, DMSO-d_6).

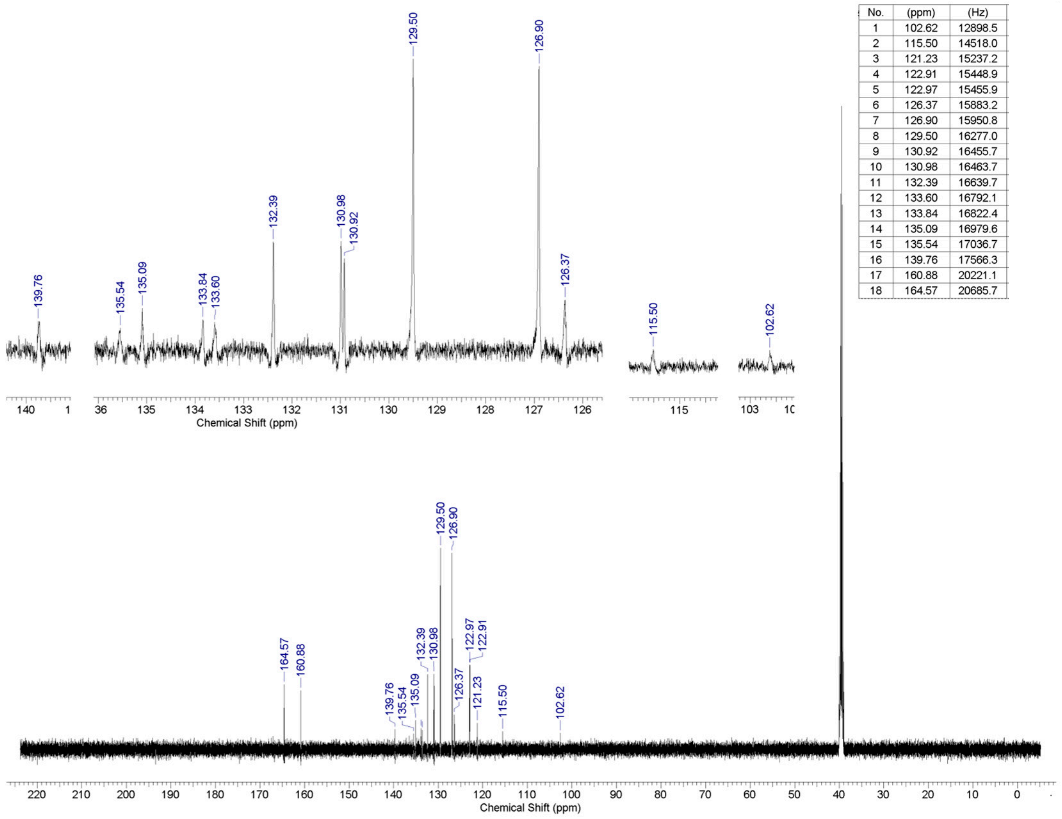

No.	(ppm)	(Hz)
1	102.62	12898.5
2	115.50	14518.0
3	121.23	15237.2
4	122.91	15448.9
5	122.97	15455.9
6	126.37	15883.2
7	126.90	15950.8
8	129.50	16277.0
9	130.92	16455.7
10	130.98	16463.7
11	132.39	16639.7
12	133.60	16792.1
13	133.84	16822.4
14	135.09	16979.6
15	135.54	17036.7
16	139.76	17566.3
17	160.88	20221.1
18	164.57	20685.7

Figure S3. ^{13}C-NMR of compound **5b** (125 MHz, DMSO-d_6).

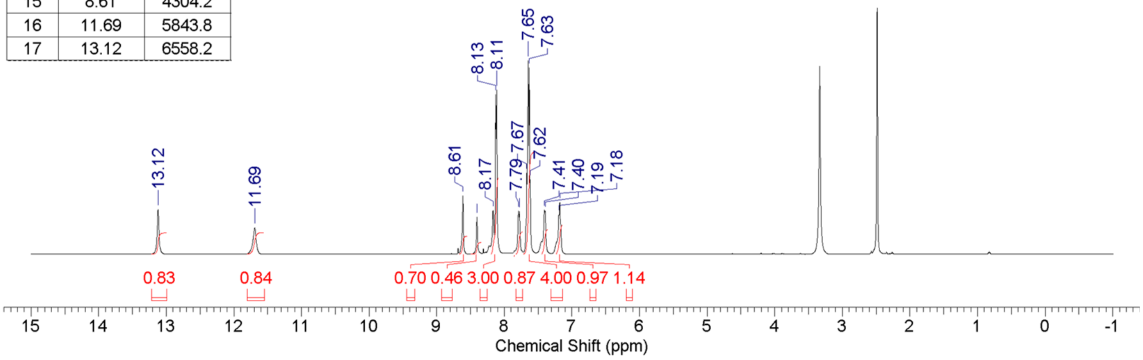

No.	(ppm)	(Hz)
1	7.18	3588.3
2	7.19	3595.1
3	7.40	3696.7
4	7.41	3702.1
5	7.62	3807.6
6	7.63	3814.4
7	7.65	3821.7
8	7.67	3832.0
9	7.78	3886.7
10	7.79	3893.5
11	8.11	4055.1
12	8.13	4062.0
13	8.17	4081.5
14	8.40	4200.7
15	8.61	4304.2
16	11.69	5843.8
17	13.12	6558.2

Figure S4. ^{1}H-NMR of compound **5c** (500 MHz, DMSO-d_6).

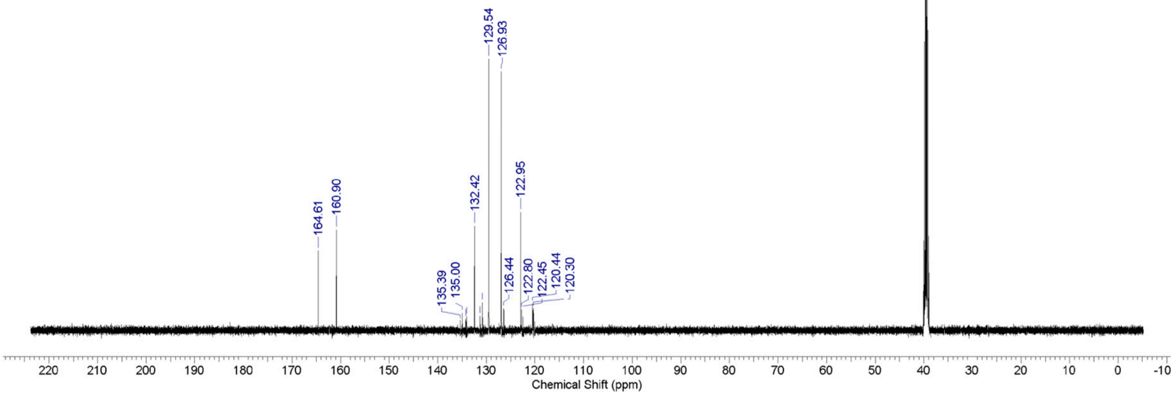

No.	(ppm)	(Hz)
1	120.30	15120.4
2	120.44	15138.4
3	122.45	15391.3
4	122.80	15435.3
5	122.95	15453.3
6	126.44	15892.8
7	126.93	15954.3
8	126.98	15960.0
9	129.54	16281.9
10	130.80	16439.9
11	131.33	16507.6
12	132.42	16644.6
13	134.07	16851.4
14	134.22	16870.7
15	135.00	16967.7
16	135.39	17017.4
17	160.90	20224.2
18	164.61	20690.1

Figure S5. ^{13}C-NMR of compound **5c** (125 MHz, DMSO-d_6).

No.	(ppm)	Hz
1	3.01	1504.3
2	3.02	1511.1
3	3.65	1823.2
4	3.66	1830.0
5	3.68	1837.3
6	6.95	3475.6
7	6.97	3482.9
8	6.98	3490.7
9	7.05	3523.5
10	7.06	3530.8
11	7.08	3538.1
12	7.20	3598.2
13	7.33	3662.2
14	7.34	3670.5
15	7.54	3769.1
16	7.56	3777.4
17	7.63	3815.5
18	7.65	3821.8
19	7.66	3829.6
20	7.67	3833.1
21	7.68	3839.9
22	7.70	3847.2
25	8.13	4061.1
24	8.14	4067.9
25	8.26	4127.5
26	8.60	4297.0
27	10.04	5019.2
28	10.89	5442.1

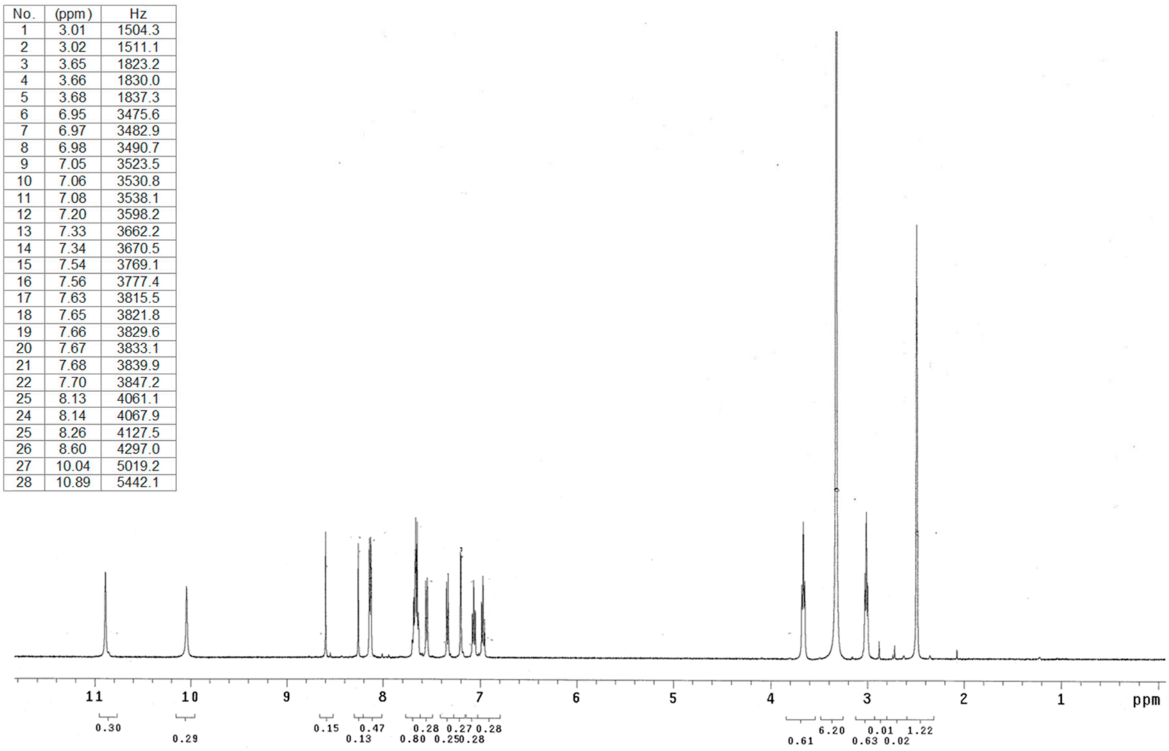

Figure S6. ^{1}H-NMR of compound **5d** (500 MHz, DMSO-d_6).

No.	(ppm)	Hz
1	7.66	1530.8
2	7.70	1539.8
3	7.74	1548.3
4	8.09	1618.5
5	8.13	1626.6
6	8.18	1635.1
7	8.37	1673.2
8	8.64	1727.7
9	11.66	2331.6
10	13.22	2643.5

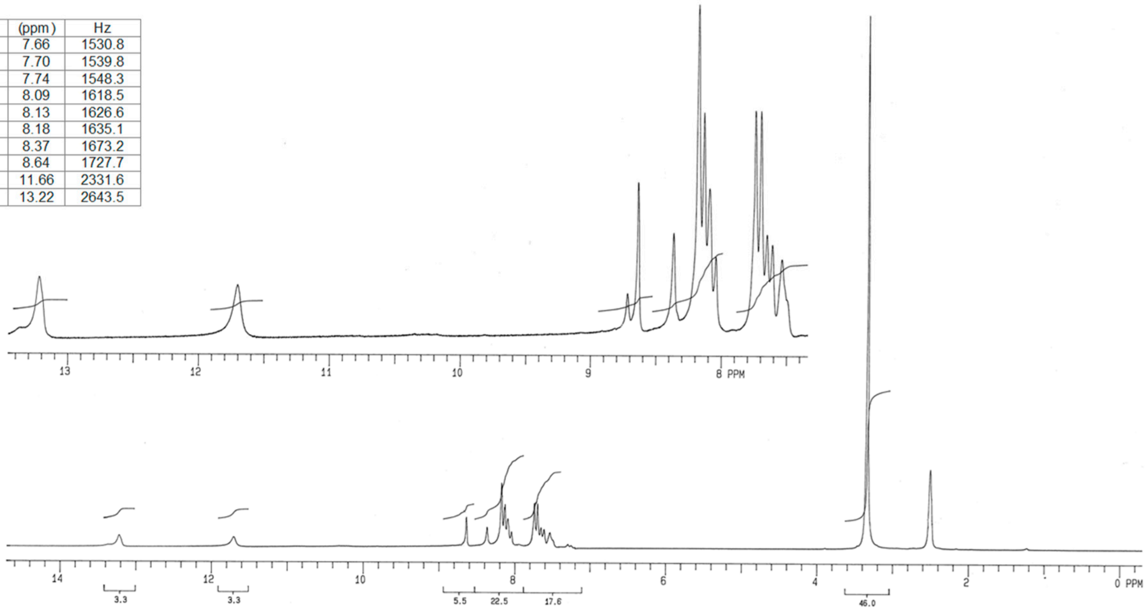

Figure S7. ^{1}H-NMR of compound **5e** (200 MHz, DMSO-d_6).

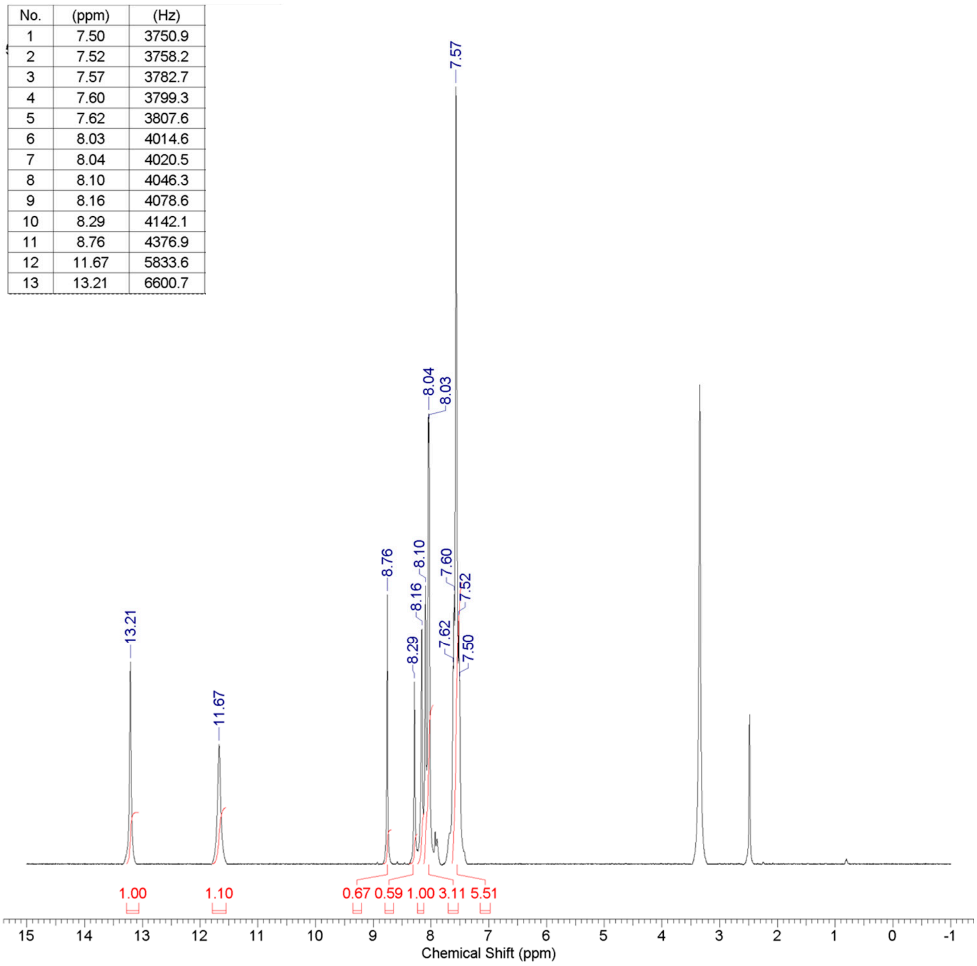

No.	(ppm)	(Hz)
1	7.50	3750.9
2	7.52	3758.2
3	7.57	3782.7
4	7.60	3799.3
5	7.62	3807.6
6	8.03	4014.6
7	8.04	4020.5
8	8.10	4046.3
9	8.16	4078.6
10	8.29	4142.1
11	8.76	4376.9
12	11.67	5833.6
13	13.21	6600.7

FigureS8. ^{1}H-NMR of compound **5f** (500 MHz, DMSO-d_6).

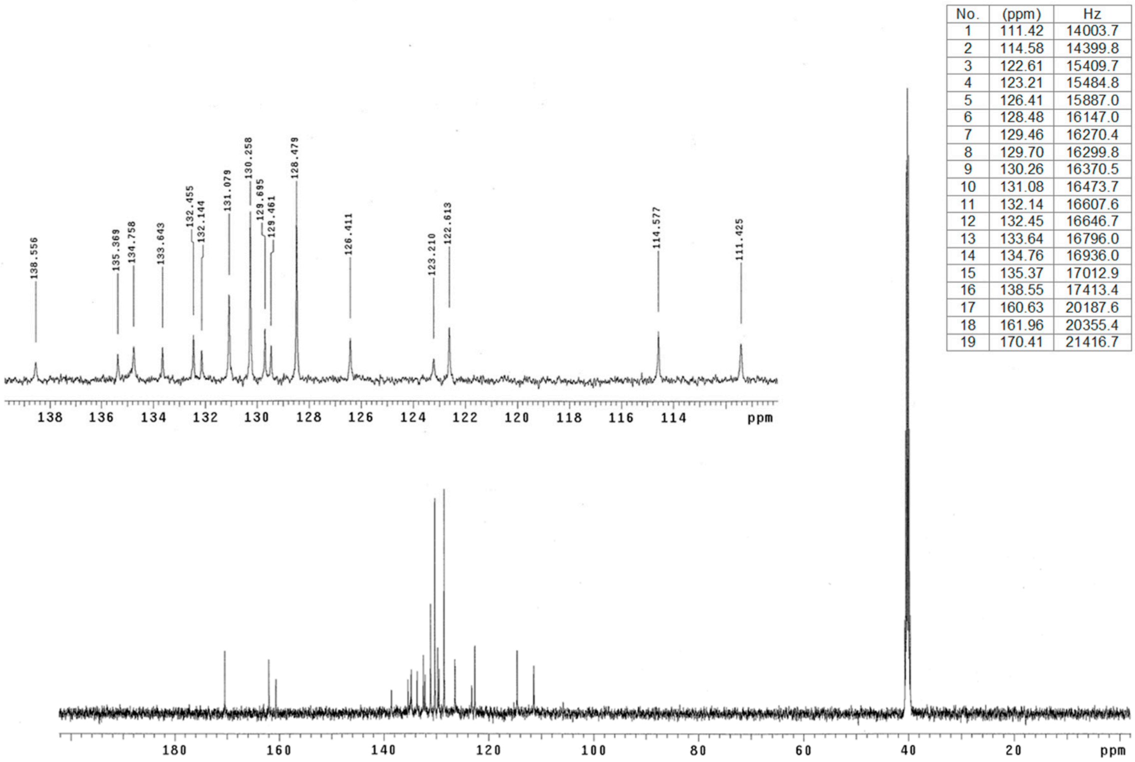

No.	(ppm)	Hz
1	111.42	14003.7
2	114.58	14399.8
3	122.61	15409.7
4	123.21	15484.8
5	126.41	15887.0
6	128.48	16147.0
7	129.46	16270.4
8	129.70	16299.8
9	130.26	16370.5
10	131.08	16473.7
11	132.14	16607.6
12	132.45	16646.7
13	133.64	16796.0
14	134.76	16936.0
15	135.37	17012.9
16	138.55	17413.4
17	160.63	20187.6
18	161.96	20355.4
19	170.41	21416.7

FigureS9. ^{13}C-NMR of compound **5f** (125 MHz, DMSO-d_6).

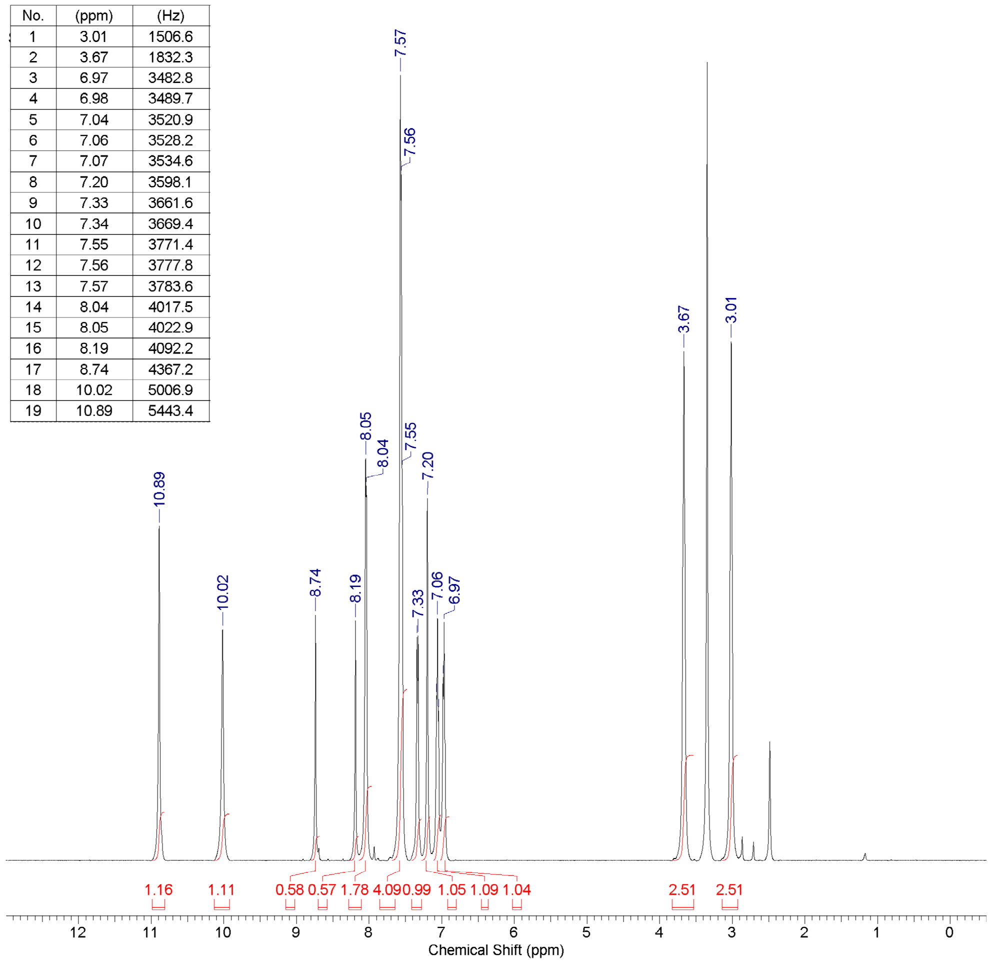

No.	(ppm)	(Hz)
1	3.01	1506.6
2	3.67	1832.3
3	6.97	3482.8
4	6.98	3489.7
5	7.04	3520.9
6	7.06	3528.2
7	7.07	3534.6
8	7.20	3598.1
9	7.33	3661.6
10	7.34	3669.4
11	7.55	3771.4
12	7.56	3777.8
13	7.57	3783.6
14	8.04	4017.5
15	8.05	4022.9
16	8.19	4092.2
17	8.74	4367.2
18	10.02	5006.9
19	10.89	5443.4

FigureS10. ^{1}H-NMR of compound **5g** (500 MHz, DMSO-*d*6).

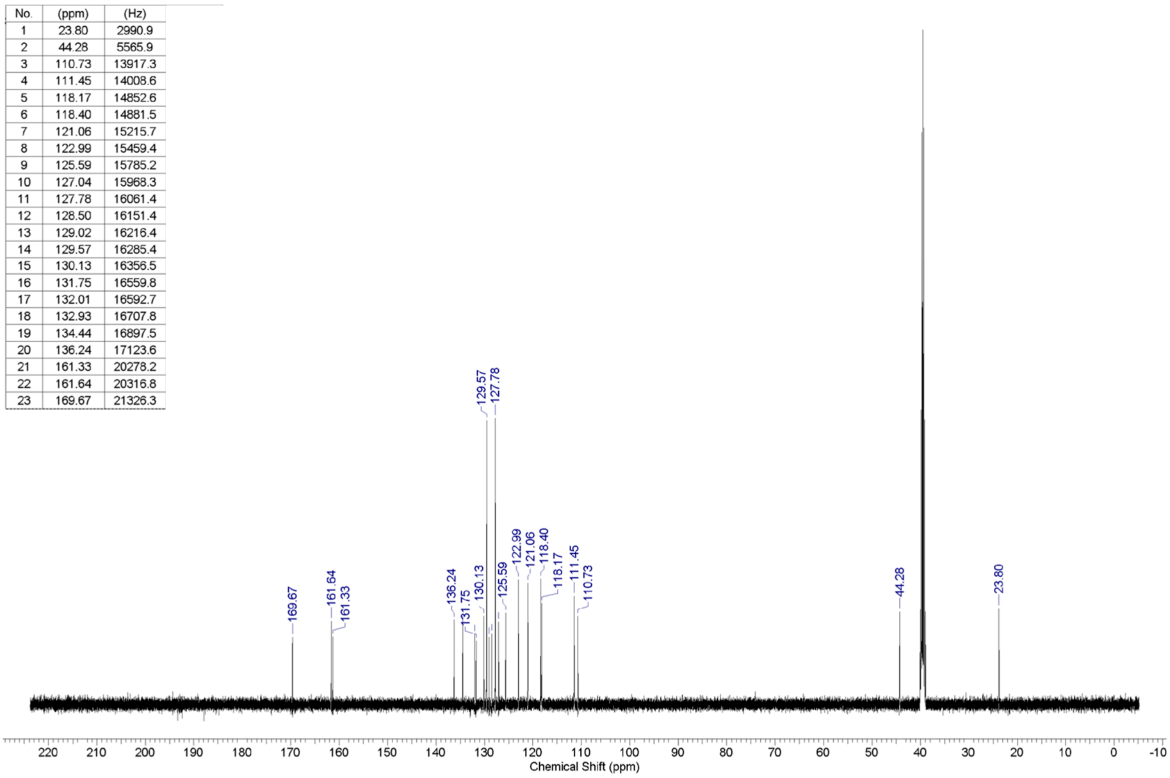

No.	(ppm)	(Hz)
1	23.80	2990.9
2	44.28	5565.9
3	110.73	13917.3
4	111.45	14008.6
5	118.17	14852.6
6	118.40	14881.5
7	121.06	15215.7
8	122.99	15459.4
9	125.59	15785.2
10	127.04	15968.3
11	127.78	16061.4
12	128.50	16151.4
13	129.02	16216.4
14	129.57	16285.4
15	130.13	16356.5
16	131.75	16559.8
17	132.01	16592.7
18	132.93	16707.8
19	134.44	16897.5
20	136.24	17123.6
21	161.33	20278.2
22	161.64	20316.8
23	169.67	21326.3

FigureS11. ^{13}C-NMR of compound **5g** (125 MHz, DMSO-*d*6).

No.	(ppm)	Hz
1	7.55	3771.5
2	7.62	3806.2
3	7.63	3813.0
4	7.64	3820.4
5	7.66	3829.2
6	8.11	4055.7
7	8.13	4066.0
8	8.15	4072.8
9	8.19	4094.3
10	8.41	4203.7
11	8.71	4352.1
12	11.75	5871.3
13	13.24	6615.9

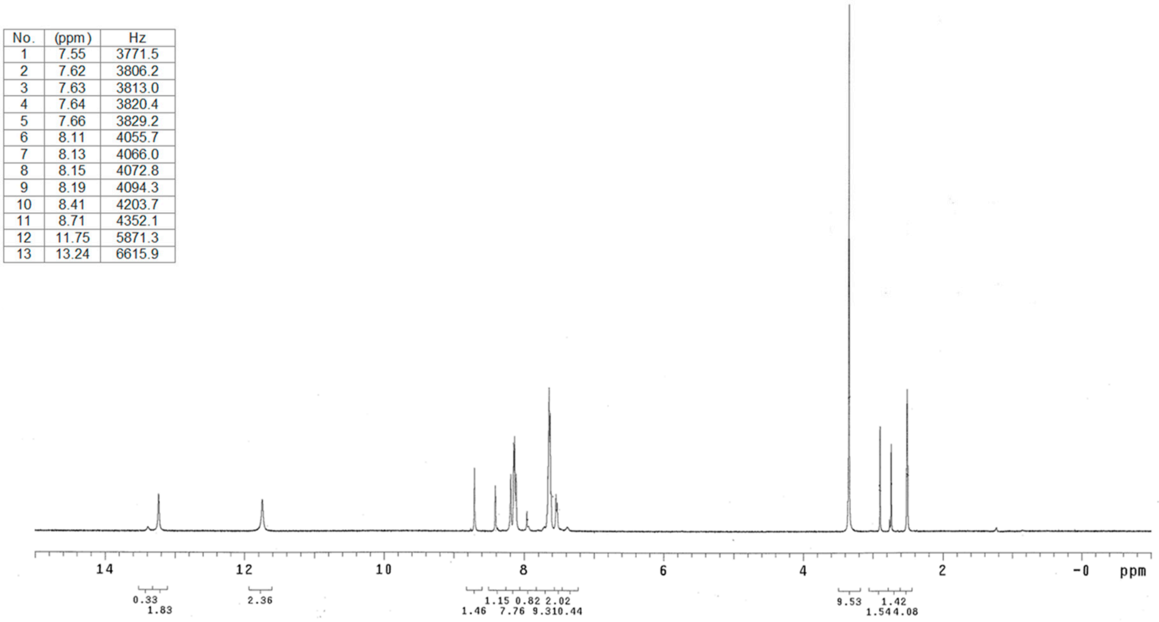

FigureS12. ^{1}H-NMR of compound **5h** (500 MHz, DMSO-d_6).

No.	(ppm)	(Hz)
1	3.00	1501.3
2	3.02	1508.6
3	3.03	1515.4
4	3.66	1828.0
5	3.67	1835.3
6	3.69	1842.1
7	6.95	3476.0
8	6.97	3483.3
9	6.98	3491.1
10	7.05	3522.4
11	7.06	3530.2
12	7.08	3537.0
13	7.20	3598.6
14	7.33	3663.0
15	7.35	3671.3
16	7.54	3769.5
17	7.56	3777.3
18	7.57	3783.1
19	7.58	3789.5
20	7.60	3797.3
21	7.62	3806.1
22	8.09	4042.0
23	8.10	4048.8
24	8.24	4116.2
25	8.64	4320.8
26	10.07	5031.8
27	10.89	5443.4

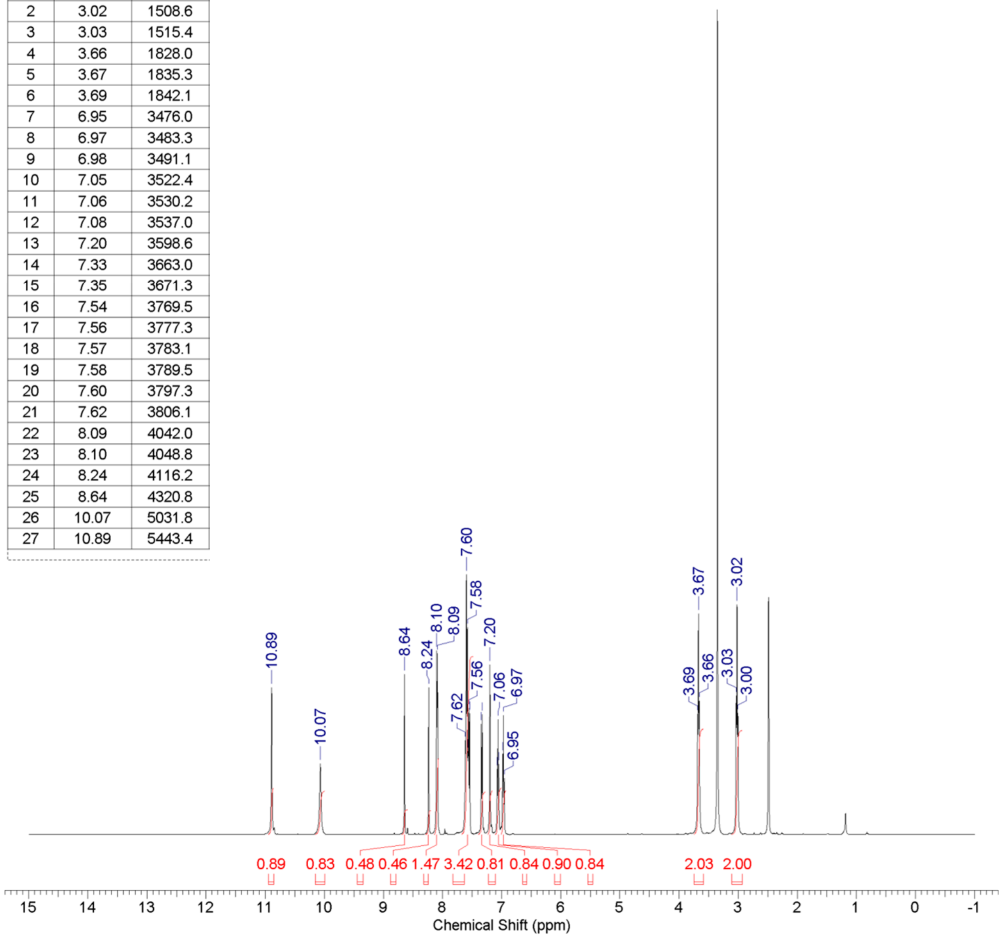

FigureS13. ^{1}H-NMR of compound **5i** (500 MHz, DMSO-d_6).

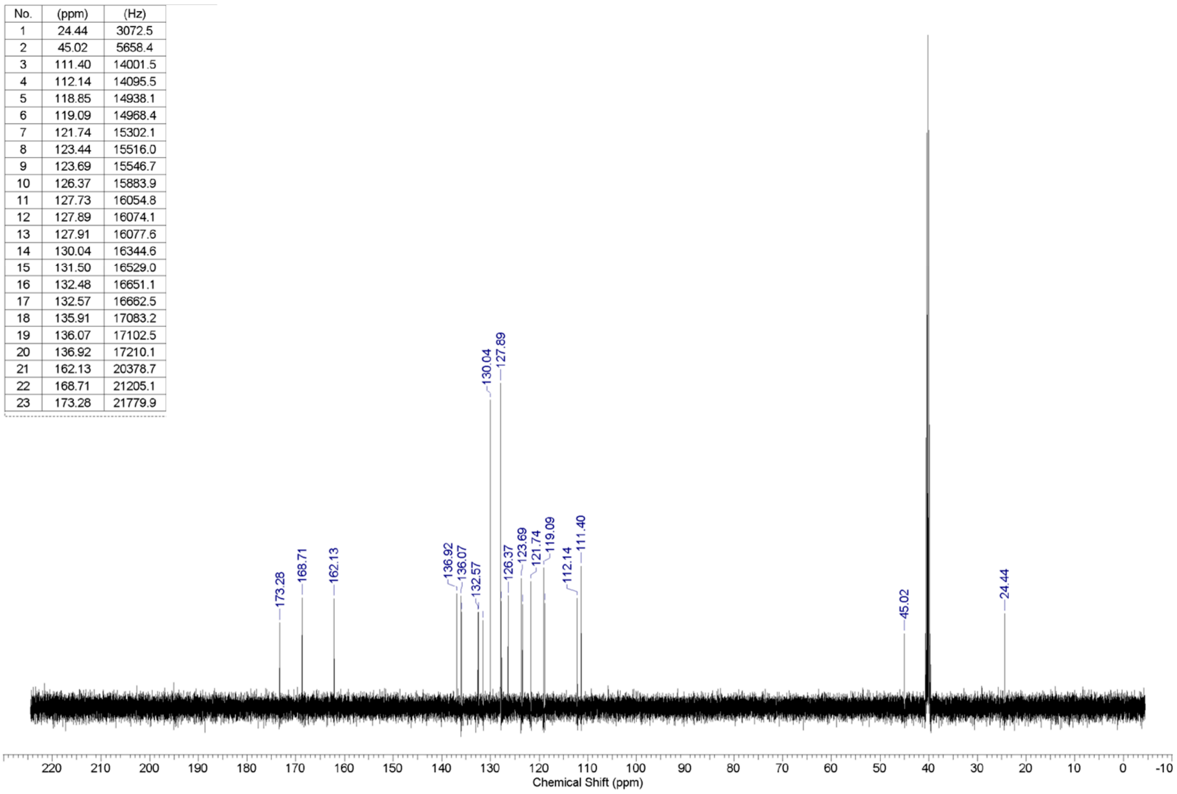

No.	(ppm)	(Hz)
1	24.44	3072.5
2	45.02	5658.4
3	111.40	14001.5
4	112.14	14095.5
5	118.85	14938.1
6	119.09	14968.4
7	121.74	15302.1
8	123.44	15516.0
9	123.69	15546.7
10	126.37	15883.9
11	127.73	16054.8
12	127.89	16074.1
13	127.91	16077.6
14	130.04	16344.6
15	131.50	16529.0
16	132.48	16651.1
17	132.57	16662.5
18	135.91	17083.2
19	136.07	17102.5
20	136.92	17210.1
21	162.13	20378.7
22	168.71	21205.1
23	173.28	21779.9

FigureS14. ^{13}C-NMR of compound **5i** (125 MHz, DMSO-d_6).

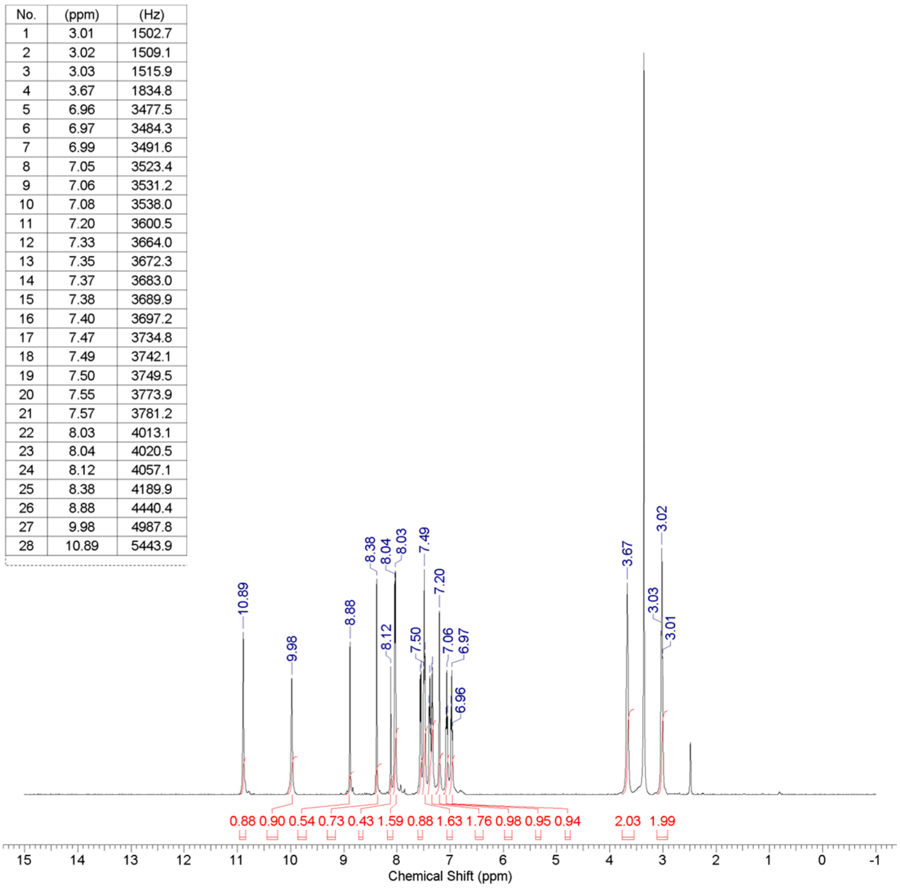

No.	(ppm)	(Hz)
1	3.01	1502.7
2	3.02	1509.1
3	3.03	1515.9
4	3.67	1834.8
5	6.96	3477.5
6	6.97	3484.3
7	6.99	3491.6
8	7.05	3523.4
9	7.06	3531.2
10	7.08	3538.0
11	7.20	3600.5
12	7.33	3664.0
13	7.35	3672.3
14	7.37	3683.0
15	7.38	3689.9
16	7.40	3697.2
17	7.47	3734.8
18	7.49	3742.1
19	7.50	3749.5
20	7.55	3773.9
21	7.57	3781.2
22	8.03	4013.1
23	8.04	4020.5
24	8.12	4057.1
25	8.38	4189.9
26	8.88	4440.4
27	9.98	4987.8
28	10.89	5443.9

FigureS15. ^{1}H-NMR of compound **5j** (500 MHz, DMSO-d_6).

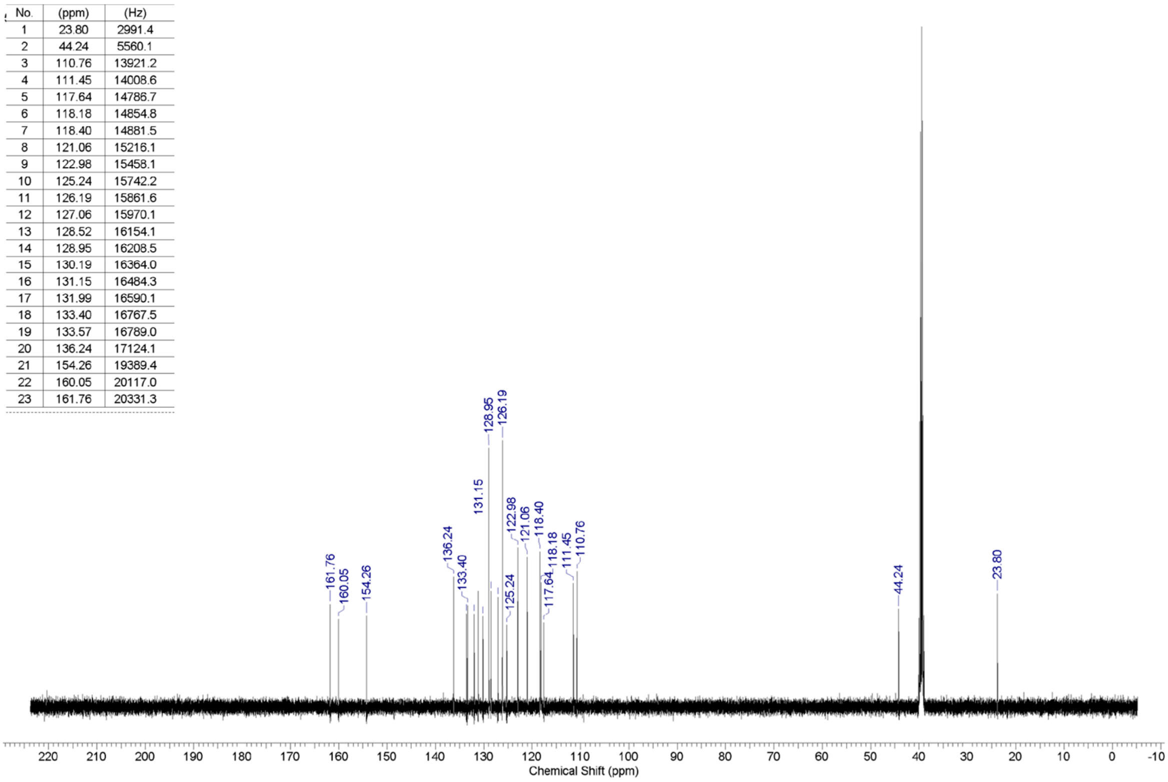

No.	(ppm)	(Hz)
1	23.80	2991.4
2	44.24	5560.1
3	110.76	13921.2
4	111.45	14008.6
5	117.64	14786.7
6	118.18	14854.8
7	118.40	14881.5
8	121.06	15216.1
9	122.98	15458.1
10	125.24	15742.2
11	126.19	15861.6
12	127.06	15970.1
13	128.52	16154.1
14	128.95	16208.5
15	130.19	16364.0
16	131.15	16484.3
17	131.99	16590.1
18	133.40	16767.5
19	133.57	16789.0
20	136.24	17124.1
21	154.26	19389.4
22	160.05	20117.0
23	161.76	20331.3

FigureS16. ^{13}C-NMR of compound **5j** (125 MHz, DMSO-d_6).
